# Enhancing the Performance of PVDF/GO Ultrafiltration Membrane via Improving the Dispersion of GO with Homogeniser

**DOI:** 10.3390/membranes12121268

**Published:** 2022-12-15

**Authors:** Xin Sun, Hana Shiraz, Riccardo Wong, Jingtong Zhang, Jinxin Liu, Jun Lu, Na Meng

**Affiliations:** 1 School of Environmental Engineering, Xuzhou University of Technology, Xuzhou 221018, China; 2 Department of Chemical Engineering, Monash University, Melbourne, VIC 3800, Australia

**Keywords:** homogeniser, PVDF, GO nanosheets, ultrafiltration membrane

## Abstract

In this study, PVDF/GO-h composite membranes were synthesised using a homogeniser to improve the dispersion of GO nanosheets within the composite membrane’s structure, and then characterised and contrasted to PVDF/GO-s control samples, which were synthesised via traditional blending method-implementing a magnetic stirrer. By characterizing membrane via X-ray diffraction (XRD), Fourier transform infrared spectroscopy (FTIR), scanning electron microscopy (SEM), water contact angle (WCA) and membrane performance. SEM results showed that the number of the finger-like structure channels and pores in the sponge like structure of PVDF/GO-h composite membranes become more compared with PVDF/GO-s membranes. Water contact angle tests showed that the PVDF/GO-h composite membranes have lower contact angle than PVDF/GO-s control, which indicated the PVDF/GO-h composite membranes are more hydrophilic. Results also showed that composite membranes blended using homogeniser exhibited both improved water flux and rejection of target pollutants. In summary, it was shown that the performance of composite membranes could be improved significantly via homogenisation during synthesis, thus outlining the importance of further research into proper mixing.

## 1. Introduction

Ultrafiltration (UF) processes have received increased attention in liquid separation in the past several decades, especially in wastewater treatment, medical, food, chemical and biochemical fields [1]. Compared to micro filtration membrane, ultrafiltration membranes prepared by ultrafiltration technology have a smaller surface pore size, between 1 and 100 nm [2], and can remove macromolecular organic matter (protein, bacteria), colloids, suspended solids [3], which makes ultrafiltration membranes play a key role in protein purification and separation.

Based on its excellent chemical resistance, antioxidation activity, thermal stability and membrane forming properties, polyvinylidene fluoride (PVDF), a semi-crystalline material, is used as an UF membrane in wastewater treatment [4,5,6,7]. However, due to the inherent hydrophobicity of PVDF material, the membrane prepared by PVDF often has serious membrane contamination, which is caused by the physical or chemical interaction between the membrane surface and the macromolecules or microorganisms in the separation solution during the membrane separation process [8]. Based on the hydrophobicity of PVDF, PVDF films tend to have a higher scaling tendency than hydrophilic films with similar separation characteristics and pore size [9]. An effective approach to solving this problem is to integrate nanomaterials into the PVDF membrane. The PVDF ultrafiltration membrane prepared by H. Younas et al. [10] by adding inorganic TiO_2_ nanoparticles (NPs) has good hydrophilicity and flux, and also has a high rejection rate of humic acid (HA). In addition, the study showed that the PVDF hybrid membrane containing vermiculite nanoparticles (Verm NPs) was prepared by the opposite method, which had higher anti-pollution performance [11]. These results indicate that the hydrophilic, permeable and antifouling properties can be improved by incorporating organic materials into PVDF polymers [12].

Graphene oxide (GO)surface contains rich groups, such as carboxyl group, hydroxyl group and epoxy group [13], which makes GO have good hydrophilicity. In addition, GO also has good mechanical strength, electrical conductivity, alkaline resistance and other excellent physical and chemical properties. Due to its excellent properties, graphene oxide is widely used in membrane separation. Therefore, graphene oxide and its derivates, as a nanofiller, may be preferred over other nanofillers owing to high aspect ratio, hydrophilicity, tensile strength, thermal conductivity and electrical conductivity [9,14,15,16]. The superior properties of graphene compared to polymers are also reflected in polymer/graphene nanocomposites membranes [4,12,17]. It was reported that the performance of polymer membrane was enhanced after GO was embedded [1,3,18,19,20,21,22,23,24,25]. Most polymer/GO hybrid membranes are prepared via an electronic stirring [1,3,18,19,20,21,22,23,24,25,26,27,28]. However, GO sheets can only be dispersed in aqueous media, which is incompatible with most organic polymers; this kind of polymer/GO membranes face the problem of poor distribution of graphene oxide into polymer [4,20,21,29]. The improvement in the properties of the nanocomposites depends on the distributions of graphene oxide layers in the polymer matrix as well as interfacial bonding between the graphene oxide layers and polymer matrix [4]. Therefore, the good distribution of GO nanosheets in the polymer matrix is of great significance to improve the performance of hybrid membranes.

Homogeniser is a commonly used mechanical method to reduce the particle in material field [30,31,32,33,34,35]. For example, Long et al. [34] used a high-speed homogeniser to treat the microcrystalline cellulose (MCC). Scanning electron microscope (SEM) results showed that the particle size of the MCC was reduced from micrometre scale down to nanoscale. Sun-Young et al. [32] prepared cellulose nanofibrils by employing a high-pressure homogenizer, and SEM results showed that the complete fibrosis of the bulk cellulose fibrils to nanoscale with high aspect ratio was accomplished by homogenization process. T. J. Nacken et al. [36] used a high pressure homogeniser to produce graphene and few layers of graphene (FLG) in a mixture of methyl pyrrolidone and water-surfactant. It was found that the high pressure homogeniser could obtain a high enough concentration of FLG suspension with low defect concentration. To the best of our knowledge, no previous study has been conducted to prepare GO/PVDF hybrid membranes using a homogeniser to disperse GO nanosheets with PVDF. Therefore, this study aims to fill this research gap.

This study aimed to fabricate a high performance PVDF/GO membrane with better GO distribution via using a homogeniser. For comparison, PVDF/GO hybrid membranes were fabricated by both conventional magnetic stirring method (PVDF/GO-s) and homogeniser dispersing method (PVDF/GO-h) and compared with PVDF membrane. XRD and FTIR analysis were conducted to ensure GO nanosheets were successfully incorporated into PVDF membranes. The WCA, water flux and rejection of hybrid membranes were tested to study the effect of GO distribution on the performance of hybrid membranes. The results of this study could shed light on the synthesis of nanomaterials incorporated membranes, which have promising application in liquid separation.

## 2. Experimental Section

### 2.1. Materials

Natural graphite power, sodium nitrate (NaNO_3_), sulfuric acid (H_2_SO_4_), potassium permanganate (KMnO_4_), hydrogen peroxide (H_2_O_2_) and hydrochloric acid (HCl; 32%) were purchased from Sigma Aldrich (St. Louis, MI, USA). PVDF (FR-904) was obtained from Shanghai 3 F new materials Co., Ltd. (Shanghai, China). The molecular weight (Mw) of PVDF is approximately 1.02 × 10^6^ g/mol, measured by GPC (waters, 515). N,N-Dimethylacetamide (DMAc) and polyethylene glycol (PEG; with MW of 3,535,000 g/mol) were purchased from Sigma Aldrich (St. Louis, MI, USA).

### 2.2. Preparation of GO Nanosheets, PVDF, PVDF/GO-s and PVDF/GO-h Homogenised Membranes 

#### 2.2.1. Preparation of GO Nanosheets

Graphene oxide (GO) was prepared using the modified Hummers’ method from graphite powder. The synthesis procedure was reported in our previous research [37]. Briefly, NaNO_3_ (1.25 g) and natural graphite (2.5 g) were first mixed in an ice water bath. Then 60 mL of sulfuric acid was added. After 30 min, 7.5 g of KMnO_4_ was added into the mixture. After that, the ice water bath was removed, and the mixture was further stirred overnight at room temperature. Subsequently, 135 mL of deionised (DI) water and 25 mL of H_2_O_2_ were added in sequence. A bright yellow mixture was obtained after the solution cooled down. GO nanosheets were obtained after the mixture was washed several times. The obtained GO nanosheets are consistent with the results of previous experiments [38].

#### 2.2.2. Preparation of PVDF Membranes

PVDF membranes were synthesised via a phase inversion method following the these steps: 3 g PVDF powder and 17 g of DMAc solution were added into a 25 mL glass vial. Afterwards, the mixture was stirred for approximately 24 h (overnight) on a magnetic stirrer in a 50 °C oil bath. When the casting solution was fully dissolved, the vial was taken out from the oil bath and allowed to rest at room temperature for another 12 h to remove the bubbles within the solution. Then, the PVDF casting solution was cast on a clean and oven-dried glass plate by using a casting knife (Elcometer 3580) with a gap of 200 μm. The whole composite was then immediately immersed in a coagulation bath of water and allowed to sit for 15 min to enable phase inversion to occur. Subsequently, the support membrane was transferred into deionised (DI) water before further use.

#### 2.2.3. Preparation of PVDF/GO-s and PVDF/GO-h Membranes

The synthetic procedure of PVDF/GO hybrid membrane was the same as that of PVDF membrane, except that 0.03 g GO was added into 3 g PVDF powder and 17 g DMAc solution to form 0.15 wt% GO casting solution. The GO-containing solution was stirred for 24 h on a magnetic stirrer in a 50 °C oil bath to completely disperse GO into the casting solution and then was allowed to rest at room temperature for another 12 h. For PVDF/GO-h membranes, after stirring for 24 h, the solution was homogenised by a homogeniser (AD500S-H) for another 5 min at 2000 rpm to further disperse GO nanosheets. And then the PVDF/GO-h solution was allowed to rest at room temperature for at least two days or until all bubbles within the solution have disappeared. Finally, both solutions were casted on glass plates to allow phase inversion to occur.

### 2.3. Characterization of PVDF and PVDF/GO Membranes

The functional groups and structure of PVDF and PVDF/GO membranes were characterised by powder X-ray diffraction (PXRD; Rigaku Mini Flex, Cu Ka radiation, Tokyo, Japan) and Fourier transform infrared spectrometer (FTIR spectrometer, PerkinElmer, Waltham, MA, USA). The surface and cross section morphologies of membranes were examined by a field emission scanning electron microscopy (FESEM; Magellan 400, Nova Nano SEM 450, FEI, New York, NY, USA). Membrane hydrophilicity was analysed via contact angle measurements (OCA-15EC, Dataphysics, Stuttgart, Germany). The static contact angle of different polymerised films was tested by the suspension drop method. After drying the film to be tested, it was flatly pasted on the slide, and then placed on the test table at room temperature. One microliter of deionised water was dropped onto the membrane surface with a microinjector. The contact angle was measured after the water drop stabilised. At least 10 contact angles at different places for each membrane were averaged to obtain a reliable value.

### 2.4. Membrane Performance Evaluation

#### Membrane Permeability and Salt Rejection

Membrane performance testing was conducted using a dead-end filtration (DEF) system (effective area is 14.2 cm^2^). The detailed filtration process was as following: (1) The membrane was first compacted at 2 bars for 3 h to achieve a steady flux; (2) the trans-membrane pressure was reduced to 1 bar and the pure water flux was recorded every 1 min. At least 60 measurements were collected to obtain an average flux value; (3) The DI water was replaced by a PEG feed solution, filtration cells were stirred at 400 rpm using a stir to minimise concentration polarization and the trans-membrane pressure was returned to 1 bar. After 1 h of filtration, a sample of the permeation solution was collected. For each membrane performance evaluations, at least three samples were tested. A total organic carbon (TOC) analyser was used to determine the concentration of PEG in the feed and permeation solution; the analyser uses combustion catalytic oxidation method at 680 °C. The rejection was calculated by the following Equation (1):

(1) $$R=(1-{\mathrm{TOC}}_{filtrate\;\;}/{\mathrm{TOC}}_{feed})\times 100\%$$

where TOC*_filtrate_* is the TOC concentration of PEG in the filtrate and TOC*_feed_* is the TOC concentration in the PEG feed solution.

## 3. Results and Discussion

### 3.1. Characterisation of PVDF, PVDF/GO-s and PVDF/GO-h Membranes

#### 3.1.1. FTIR

**Figure 1:**
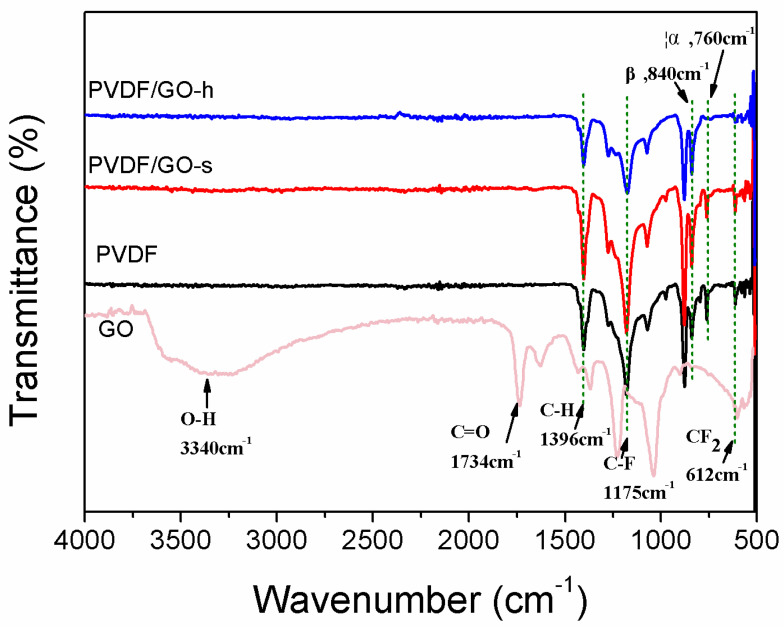
FTIR spectra of PVDF, PVDF/GO-s and PVDF/GO-h membranes.

Figure 1 shows the FT-IR spectra of PVDF, PVDF/GO-s and PVDF/GO-h membranes and GO. As can be seen in Figure 1, pristine PVDF membrane shows peaks at 1396 cm^−1^ and 1175 cm^−1^, attributing to C-H and C-F stretching and deformation [39], which are also prominent in all other composite membranes. The prominent features of the GO spectrum is the adsorption peaks at ~3340 cm^−1^ and ~1734 cm^−1^, which are corresponding to O-H and C=O stretching vibrations, respectively [4]. Due to the nucleation effect of nano-filler in the PVDF matrix [40], it can be noticed that the intensity of α phase (at 760 cm^−1^)decreases when GO is embedded in the PVDF matrix, while the intensity of β phase (at 840 cm^−1^) increases. This indicates that GO nanosheets contain sufficient carbonyl groups to nucleate most of PVDF chains into β-phase.

Yu et al. calculated the absorption energy of α-and ß-polyform [41], and found that the significant difference in the adsorption energy of α and β phase made the energy barrier between trans-gauche-trans-gauche0 (TGTG0) and trans-trans (TT) structure increase, and it became difficult to convert TGTG0 to TT structure in the process of polymer crystallization. It can be seen from Figure 1 that compared with PVDF/GO-s, α phase of PVDF/GO-h membrane after homogenisation process decreases or even disappears. This is because the use of homogeniser can not only improve the dispersibility of GO in solution, but also effectively reduce the adsorption energy difference between α phase and β phase, overcome the energy barrier [42], help PVDF chain adsorption to the GO surface, promote the interaction between the oxygen-containing groups on GO and the hydrogen atoms on the PVDF chain. However, the peak value of β phase of PVDF-h was lower than that of PVDF-s phase, which may be because the dispersibility of GO in solution was improved by the use of homogeniser, and thus the concentration of solution was increased. This will lead to GO as a filler particle agglomeration under high concentration, resulting in reduced PVDF chain constraint and resulting in decreased β phase content [43]. L. He et al. [44] changed the crystal distribution in PVDF by adding hyperbranched chain copolymer (HBCs) modified multi-walled carbon nanotubes, and improved the β phase and thermal stability of the membrane. We used a homogeniser to enhance the β phase of the PVDF membrane. After homogenisation, the α phase of PVDF hybrid membrane was almost completely transformed into β phase. Compared with the former, the conversion rate of α phase to β phase of PVDF hybrid membrane was greatly improved by using the homogeniser. Therefore, homogeniser can change PVDF α-phase to β-phase to a greater extent. These results are in good agreement with previous studies using other carbon materials as fillers in PVDF membranes [39,45,46].

The composite membranes show no absorption peak at ~3340 cm^−1^, which would be indicative of O-H stretching of carboxylic acid. One possibility is that because of the strong compatibility between the carbonyl group in GO and the fluorine in PVDF [47]. Another explanation is that the casting solution concentration using 0.15 wt% GO is too low for the functional groups to present at any significant level detected by FT-IR.

#### 3.1.2. XRD

**Figure 2:**
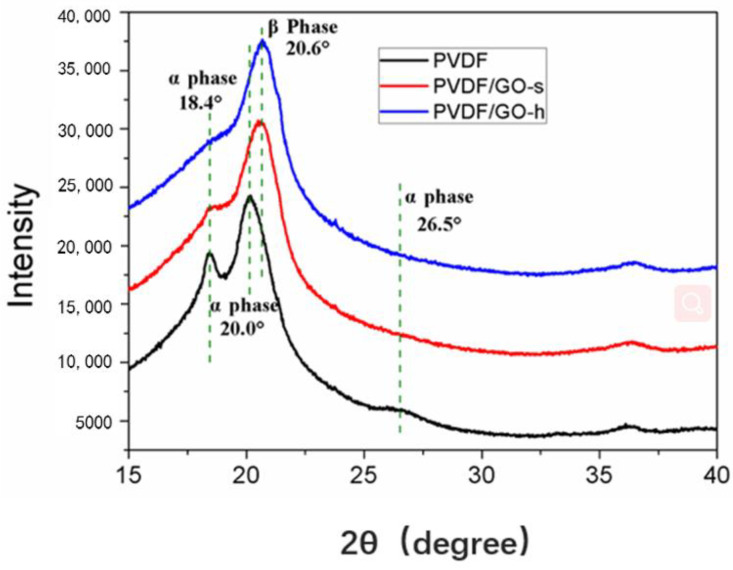
XRD spectra of PVDF, PVDF/GO-s and PVDF/GO-h membranes.

To compare the molecular structure of PVDF/GO-s membrane and PVDF/GO-h membrane and confirm that GO components were successfully integrated into the PVDF polymer matrix, X-ray diffraction was performed. Figure 2 shows the results of XRD analysis of PVDF, PVDF/GO-s, and PVDF/GO-h membranes in the range of 15° to 40°, in terms of arbitrary scale of intensity. For pristine PVDF membrane, the characteristic peaks at 18.4°, 19.9° and 26.5° can be observed, which are attributed to α-phase. Both PVDF/GO-s and PVDF/GO-h membranes display a new diffraction peak at 20.6°, which corresponds to the β-phase. This is most likely due to the crystal transformation of PVDF [48]. The formation of β-polymorph is attributed to the interaction between the CF_2_ segments in PVDF polymer and the carbonyl groups (-C=O) present in GO nanosheets [39,49]. In addition, for PVDF/GO-s membranes, the intensity of α-phase peak at 18.4° dropped and the α-phase peaks at 26.5° disappeared, however, for the PVDF/GO-h membrane, both the α-phase peaks at 18.4° and 26.5° disappeared. Thus, it can be concluded that the disappearance of α-phase in PVDF/GO-h membrane indicate the enhanced crystal transformation of PVDF membrane, which is caused by the better dispersion of GO by homogeniser.

### 3.2. Membrane Morphology

#### 3.2.1. SEM Image of the Membrane 

**Figure 3:**
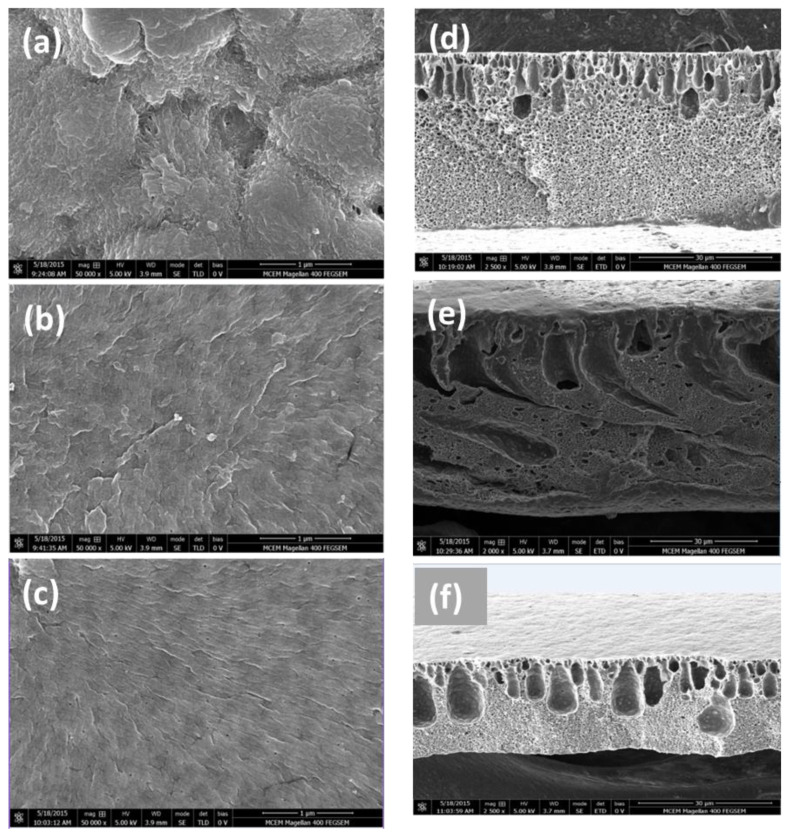
SEM images of top surface for (**a**) pure PVDF, (**b**) PVDF/GO-s, and (**c**) PVDF/GO-h membranes and images (**d**–**f**) are SEM cross sections for the same membranes, respectively.

Figure 3 displays the surface and cross-section SEM images of the pristine PVDF, PVDF/GO-s and PVDF/GO-h membranes. More SEM images obtained at the surface for the different membranes are provided in Figure 3a–c featuring the increase in the number of pores in the membrane through the addition of GO to the PVDF membrane structure.

As visible from the images in Figure 3, PVDF membrane contains clusters of pores which are large and apparent. The pore distribution is uneven and concentrated on certain areas of the membrane. The size of the pores is decreased with the addition of GO whilst the number of pores significantly increased, especially in the case of the membranes which were homogenised with GO. The reason for the GO embedded membranes showed an increase in the number of pores on the surface may be that the presence of the rich oxygen-containing functional groups which increases the rate of diffusion and thereby increases pore formation. Homogenised solutions contained more GO elements which suggests proper mixing has taken place between PVDF and GO compared to PVDF/GO prepared using a magnetic stirrer. This could explain the greater number of pores on the surface of the membrane for Figure 3c compared to Figure 3b.

As can be seen from cross-section SEM images in Figure 3d–f, the morphological changes between the GO embedded membranes and the pristine PVDF membrane were compared. All the membranes displayed a thin dense top-layer, along with a porous finger-like sublayer [50]. The skin layer is brought about as a result of the polymer concentration gradient that takes place when the membrane is immersed in the water bath immediately after preparation. The outer surface solidifies creating a dense skin layer. The fingers-like pores are created due to the phase inversion method. As this process occurs, demixing takes place between the water and the solvent, which slows down eventually due to the presence of the solid membrane. As a result of this time lag, caused by the delay, a dense yet porous sponge layer forms towards the bottom of the membrane [50,51].

For the membranes with GO added, the sub layer was visibly different. The finger-like pores in the PVDF/GO membranes were much wider than that of the pure PVDF membrane. The longer pore channels result from an increased rate of diffusion brought about by the hydrophilicity of GO. Rapid solidification from this diffusion creates wider pore channels [45]. The images further show the formation of a sponge-like cross-section for the PVDF membrane. This was not the case for the PVDF/GO membranes as the addition of GO into the structure of the membrane mostly prevented and strongly controlled the formation of this type of cross-section. The addition of GO also creates a floppy inner cross section because of the increased mass transformation that occurs between the solvent and the non-solvent during the process of phase inversion.

Compared to the cross-section of the PVDF/GO-s membranes, the finger-like structure channels in PVDF/GO-h membranes become thinner and shorter, also the pores in the sponge-like structure become smaller; however, the number of the finger-like structure channels and pores in the sponge-like structure increases. One reason may be that in the homogenisation process, the casting solution experienced extremely strong shear and thrust forces [34], the turbulence occurred in the shear gap between the rotor and stator also provided strong mixing power to the suspension, which improved the phase inversion. M. Hmamm et al. [52] found that when the crystallinity of polymer increased, the free volume would decrease correspondingly. Another possibility is that the synergistic effect of GO and PVDF is enhanced during the homogenization process, which eliminates the unique GO peak, improves the crystallinity of the PVDF hybrid film and decreases the free volume size of the hybrid film.

#### 3.2.2. Surface Hydrophilicity

**Figure 4:**
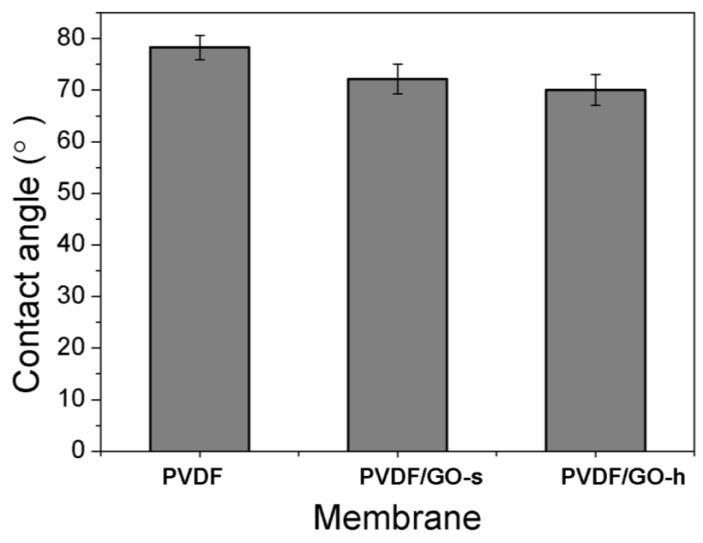
Water contact angles of PVDF, PVDF/GO-s and PVDF/GO-h membranes.

The hydrophilicity of PVDF, PVDF/GO-s and PVDF/GO-h membranes were characterised by the water contact angle. As can be seen in Figure 4, for GO embedded membranes, the water contact angle is reduced compared with pure PVDF membranes, indicating the improved hydrophilicity after GO incorporation. This could be because hydrophilic GO migrates spontaneously to the membrane/water interface to reduce the interface energy during the phase inversion process [21,50,53]. This also can be verified by the different colour between the surface and bottom, the colour of the surface is darker than the bottom. Previous research also found the same phenomenon [21,24]. The contact angle of the PVDF/GO-h membrane is slightly lower than that of PVDF/GO-s membrane, suggesting the surface of the PVDF/GO-h membrane is more hydrophilic than PVDF/GO-s membrane. The reason may be that the surface of PVDF/GO-h membrane becomes smooth, which is due to the large peaks and valleys on the surface are replaced by many smaller ones. It is also possible that GO is well dispersed in the polymer matrix after the action of the homogeniser, and the abundant oxygen-containing groups on the surface of GO can be evenly distributed on the membrane surface, thus effectively improving the surface hydrophilicity of the PVDF hybrid membrane and making the surface of the PVDF/GO-h membrane more hydrophilic than that of the PVDF/GO-s membrane.

### 3.3. Membrane Evaluation

#### 3.3.1. Membrane Flux

**Figure 5:**
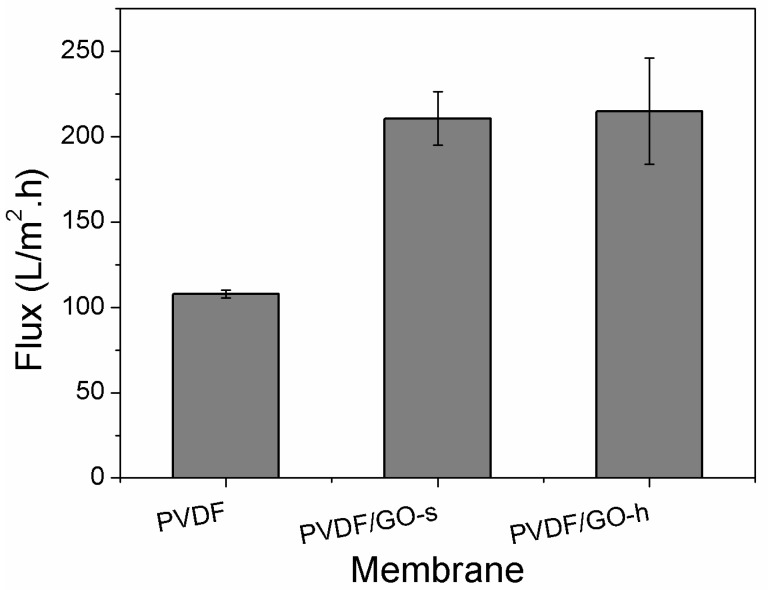
Water fluxes of the prepared PVDF, PVDF/GO-s and PVDF/GO-h membranes (200 MPa).

The permeability of the PVDF, PVDF/GO and PVDF/GO-h membranes were evaluated by measuring water flux. Figure 5 shows the water flux of these membranes. Both PVDF/GO-s membranes and PVDF/GO-h membranes exhibit higher water flux compared with the pure PVDF membrane. One reason could be due to the enhanced surface hydrophilicity after GO incorporation, as shown in the contact angle test (Figure 4). Another reason could be the enhanced phase inversion of solvent and non-solvent due to the presence of hydrophilic GO [20,24]. As shown in Figure 4, the ‘finger-like’ structure pores of PVDF/GO-s and PVDF/GO-h membranes become wider and longer.

Comparing PVDF/GO-s and PVDF/GO-h membranes, it is found that PVDF/GO-h membrane has a slightly higher flux. As shown by SEM results (Figure 3), compared with the internal structure of PVDF/GO-h membrane, the free volume of PVDF/GO-h membrane decreased, while the number of free volumes increased correspondingly. H.F.M. Mohamed et al. [54] studied the relationship between the free volume in Nafion films and the permeability of O_2_ and H_2_ and found that the larger the free volume, the better the gas permeability. This is consistent with the conclusion of another study by H.F. Mohamed et al. [55]. The decrease in free volume in the PVDF/GO-h membrane reduces the aqueous permeability of the PVDF/GO-h membrane, while the higher porosity increases the aqueous permeability, which makes the PVDF/GO-h membrane flux slightly higher than that of the PVDF/GO-s membrane.

#### 3.3.2. Membrane Rejection

**Figure 6:**
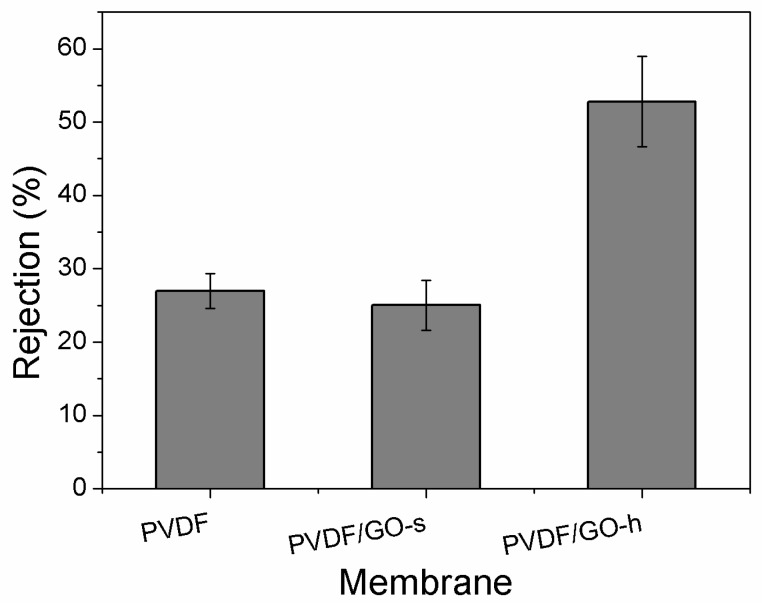
Water rejections of the prepared PVDF, PVDF/GO-s and PVDF/GO-h membranes (PEG 35 K).

To evaluate the effect of homogeniser on the membrane performance, the PEG (35 K) rejection of the hybrid membrane was measured. From Figure 6, it can be seen that the PEG rejections of pristine PVDF, PVDF/GO-s and PVDF/GO-h membranes are 26.99%, 25.02% and 52.81%, respectively. There are no significant difference of the rejection between PVDF and PVDF/GO-s membranes, it is similar as our previous research [37]. However, after using homogeniser, the PEG rejection is increased significantly from 26.99% to 52.81%. This is caused by the change of membrane morphology using the homogeniser. GO can be used as a pore-making agent to improve the number of pores in the PVDF membrane. According to the sieving principle of pore size, larger pores, such as PVDF/GO-S membrane, allow PEG molecules to pass through the membrane more easily than membranes with smaller pores on the surface (such as PVDF/GO-H membrane). After the PVDF/GO solution is homogenised by the homogeniser, the dispersibility of GO in the mixed solution is greatly improved, and the porosity of PVDF will be improved. Second, after the solution is homogenised, the oxygen-containing groups on GO surface will also be fully embedded into the PVDF membrane, the water molecules around PEG will be replaced by the hydroxyl groups on the GO surface to form a hydration layer, making the diameter of PEG larger than the diameter of the channel gap, thus improving the retention rate of PEG. It is not difficult to explain that PVDF/GO-h membrane has better interception effect on PEG than PVDF/GO-s membrane.

## 4. Conclusions

In this study, PVDF/GO-h composite membranes were synthesised using a homogeniser (AD500S-H), and results from dead-end filtration (DEF) showed that these membranes exhibited higher water flux and rejection of target pollutants compared to control samples of PVDF/GO-s prepared solely from mixing with a magnetic stirrer. This suggests that using a homogeniser disperser to mix GO into a PVDF membrane yields significantly better characteristics and should therefore be utilised as a future method for membrane preparation. Whilst these qualities are attractive in a mem-brane for use in ultrafiltration methods, results of the water contact angle test indicate that the composite membranes exhibited increased hydrophobicity as opposed to the control membranes. This implies that there is still potential for improvement of the experiment where additional parameters should be tested for or if other additives should be incorporated in the methodology to increase membrane performance.

## Data Availability

Data is contained within this article.
